# Immunogenetic Factors Affecting Susceptibility of Humans and Rodents to Hantaviruses and the Clinical Course of Hantaviral Disease in Humans

**DOI:** 10.3390/v6052214

**Published:** 2014-05-26

**Authors:** Nathalie Charbonnel, Marie Pagès, Tarja Sironen, Heikki Henttonen, Olli Vapalahti, Jukka Mustonen, Antti Vaheri

**Affiliations:** 1 INRA, UMR CBGP (INRA/IRD/Cirad/Montpellier SupAgro), Campus international de Baillarguet, CS 30016, Montferrier-sur-Lez F-34988, France; E-Mail: marie.pages@supagro.inra.fr; 2 Laboratoire de génétique des microorganismes, Université de Liège, Liège 4000, Belgium; 3 Department of Virology, Haartman Institute, University of Helsinki, POB 21, FI-00014 Helsinki, Finland; E-Mails: tarja.sironen@helsinki.fi (T.S.); olli.vapalahti@helsinki.fi (O.V.); antti.vaheri@helsinki.fi (A.V.); 4 Finnish Forest Research Institute, POB 18, FI-01301 Vantaa, Finland; E-Mail: heikki.henttonen@metla.fi; 5 Department of Virology and Immunology, HUSLAB, Helsinki University Central Hospital, FI-00014 Helsinki, Finland; 6 Department of Veterinary Biosciences, Faculty of Veterinary Medicine, University of Helsinki, FI-00014 Helsinki, Finland; 7 School of Medicine, University of Tampere, FI-33521 Tampere, Finland; E-Mail: jukka.mustonen@uta.fi; 8 Department of Internal Medicine, Tampere University Hospital, FI-33521 Tampere, Finland

**Keywords:** hantavirus, *Puumala* virus, interaction, hosts, reservoirs, rodents, immunity-related genes

## Abstract

We reviewed the associations of immunity-related genes with susceptibility of humans and rodents to hantaviruses, and with severity of hantaviral diseases in humans. Several class I and class II *HLA* haplotypes were linked with severe or benign hantavirus infections, and these haplotypes varied among localities and hantaviruses. The polymorphism of other immunity-related genes including the *C4A* gene and a high-producing genotype of *TNF* gene associated with severe PUUV infection. Additional genes that may contribute to disease or to PUUV infection severity include non-carriage of the interleukin-1 receptor antagonist (*IL-1RA*) allele 2 and *IL-1β* (-511) allele 2, polymorphisms of plasminogen activator inhibitor (*PAI-1*) and platelet *GP1a*. In addition, immunogenetic studies have been conducted to identify mechanisms that could be linked with the persistence/clearance of hantaviruses in reservoirs. Persistence was associated during experimental infections with an upregulation of anti-inflammatory responses. Using natural rodent population samples, polymorphisms and/or expression levels of several genes have been analyzed. These genes were selected based on the literature of rodent or human/hantavirus interactions (some *Mhc* class II genes, *Tnf* promoter, and genes encoding the proteins TLR4, TLR7, Mx2 and β3 integrin). The comparison of genetic differentiation estimated between bank vole populations sampled over Europe, at neutral and candidate genes, has allowed to evidence signatures of selection for *Tnf*, *Mx2* and the *Drb Mhc* class II genes. Altogether, these results corroborated the hypothesis of an evolution of tolerance strategies in rodents. We finally discuss the importance of these results from the medical and epidemiological perspectives.

## 1. Introduction

### 1.1. Immunogenetics and Diseases

It is now well established that host genetic variation influences individual susceptibility to infectious as well as autoimmune diseases [1]. The field of immunogenetics is at the core of research aiming at identifying and understanding associations between genetic factors and immunological phenotypes or immunity-related diseases [2]. Firstly based on candidate gene approaches, immunogenetics has now moved towards genomics with the advent of new technologies including DNA microarrays and next-generation sequencing. Whole genome sequencing of individuals with extreme phenotypes of infectious diseases and subsequent genome-wide association studies are now contributing to reveal the genetic bases of human susceptibility to particular infectious diseases and to decipher the immunological mechanisms underlying the pathogenesis of these diseases see for reviews [1,3,4]. Although similar research has been carried out on infectious diseases of domestic animals e.g., [5,6], the application of immunogenetics to wild animals, which constitute a large part of the vectors/reservoirs of agents of zoonotic diseases [7], remains scarce. It mainly focused on genes of the major histocompatibility complex (*Mhc*, equivalent of *Hla*—*human leukocyte antigen*—in humans) until the need for more candidate immune target genes had been pointed out [8]. For example, gene candidate approaches related to innate immunity e.g., toll-like receptors or cytokines, [9,10] as well as genomic approaches [11,12,13] have recently been developed on wild birds, fishes and rodents to evaluate the influence of molecular mechanisms on susceptibility to infectious diseases. Investigating spatio-temporal variations of allele/single nucleotide polymorphism (SNP) frequencies at these genes/loci provided further insight into the potential role of these polymorphisms in the susceptibility to infectious diseases [14], the epidemiological consequences of this variability [15] and the evolutionary mechanisms (selection, migration, drift) maintaining immune gene diversity [16,17]. Strikingly, only recently has this evolutionary perspective been explored in human studies [18].

### 1.2. Hantavirus Infection and Disease

Humans are “incidental hosts” for hantaviruses and are typically infected via contaminated aerosolized secretions (feces, urine, saliva) of the reservoir animals, which mainly include rodents, but also shrews, moles and bats though no human connection has been established yet with hantaviruses from the three latter host groups. The clinical course of human hantaviral infections varies greatly according to the different hantaviruses, ranging from no disease to mild course and low case-fatality rate (0.1% in *Puumala* virus –PUUV– infection) to severe course up to 40%–50% in *Sin Nombre* –SNV– and *Andes* –ANDV– virus infections [19,20]. In addition, large variation in clinical severity exists among patients for a given hantavirus species. Serological surveys conducted in Europe and in the Americas have demonstrated the presence of antibodies in humans who had no history of clinical disease with hemorrhagic fever with renal syndrome (HFRS) or hantavirus cardiopulmonary syndrome (HCPS) [19,21]. This suggests that even for some human pathogenic hantaviruses, some infections could be subclinical. More specifically, it is known that the course of nephropathia epidemica (NE), a mild form of HFRS in patients infected with PUUV is highly variable, ranging from asymptomatic [22] to occasionally fatal disease [23]. Hypotension up to clinical shock are for example present in less than 10% of the hospitalized patients, 5% may require dialysis, while some fatal outcomes have been reported less than 0.1% [24,25]. Although complex interactions are likely to underlie this variability, the importance of host genetics in the susceptibility to hantavirus infections and in the severity of the disease has begun to gain evidence.

In rodents, which are the reservoirs of pathogenic hantaviruses, infection is persistent [26,27] and mainly asymptomatic but see [28,29,30]. Nevertheless rodents differ in their probability of being infected with their associated hantavirus e.g., [31]. Experimental infections have confirmed that the outcome of a given hantavirus infection could vary among rodents [32,33]. As in humans, the genetic background of the reservoirs could mediate this variability.

### 1.3. Potential Applications

We review the studies on associations between immunity-related gene variation (coding sequence and levels of transcription) and the outcomes of hantavirus infection, considering both the probability of getting infected and the severity of the diseases, in humans and rodents. These results are of major medical importance because they can help predicting disease progression in hospitalized patients and can lead to better therapeutics and vaccines. Furthermore, they may improve our understanding of the epidemiology of hantaviruses, by providing a more precise comprehension host switching and ultimately hantavirus transmission from reservoirs to humans.

## 2. Impact of Genetic Factors in Human Hantavirus Infections

### 2.1. Sequence Polymorphism of Immunity-Related Genes and the Severity of Human Hantavirus Infections

Gene candidate approaches have been developed to emphasize associations between human genotypes and the clinical severity of hantavirus infections, with the aim of deciphering the genetic factors that have a major influence on the outcome of these infections. Immunogenetic investigations have mainly focused on the human leukocyte antigen (*HLA*) system, and on genes encoding molecules associated with this complex such as the C4A component of the complement system. Few other additional genes have been investigated. We detail the results of these studies below (see Table 1 for a summary).

*HLA system.* It encompasses 224 genes in a 3.6-Mb region of chromosome 6 in humans [34]. It is an essential component of the immune system with about 39.8% of these genes being immunity-related ones. Forty of the total genes belonging to the *HLA* system encode leukocyte antigens. The role of these cell-surface antigens is to present pathogen-derived antigens to T cells and to initiate acquired immune responses [35]. Some of these genes (*i.e.*, class I and class II genes) are among the most polymorphic in humans. For example, more than 100 allelic variants have been identified in human populations at the *HLA-B* and HLA-DRB1 loci (IMGT/HLA database, [36]). Many associations between alleles or combinations of alleles and susceptibility to infectious and autoimmune diseases have been described in humans e.g., [4,37].

For HFRS and HCPS, risk *HLA* haplotypes have been identified according to the following clinical and laboratory parameters of disease severity: treatment time at hospital (overall severity), weight change during hospital care (amount of fluid retention during oliguric phase), need of dialysis, lowest systolic blood pressure, presence of shock, increase of plasma creatinine and urea (severity of acute kidney injury—AKI), decrease of platelets (thrombocytopenia) and increase in blood leukocyte count (leukocytosis) [19]. There is a great geographic variability in the *HLA* alleles and haplotypes associated with hantavirus disease severity, both for *HLA* class I (*HLA-B*) and class II (*HLA-DRB*) genes.

In Finland, the individuals with *HLA* alleles HLA-B*08 and DRB1*0301 are likely to have the most severe form of the PUUV infection with lower blood pressures, higher creatinine [38] and more virus excretion into the urine and into the blood [39]. On the contrary, individuals with HLA-B*27 have a benign clinical course [40]. In Slovenia, the HLA-DRB1*15 haplotype was more frequent in patients with severe PUUV-HFRS progression than in patients with a mild course of the disease [41].

In China, the most severe HFRS cases due to Hantaan virus (HNTV) were associated with the presence of HLA-B*46 allele and HLA-B*46–DRB1*09 or HLA-B*51–DRB1*09 haplotypes [42]. In contrast, the HLA-DRB1*12 allele was more frequent in patients with a mild form of the disease but this relation was only marginally significant [43].

In the USA, the HLA-B*3501 and HLA-DRB1*1402 alleles are associated with increased risk of severe Sin Nombre (SNV)-induced HCPS [43,44,45]. In another study, HLA-B*35-restricted memory T-cell responses were related to mild disease outcome in HCPS due to Andes virus [46]. In the Chilean population, HLA-DRB1*15 was associated with a mild form of HCPS due to Andes virus whereas HLA-B*08 was again correlated with the severe course of this disease [47].

**Table 1:** Immunity-related genes associated with the severity of hantavirus disease in humans.

	Gene	Country	Haplotype	Expression	Hantavirus	Relevance with Disease Severity (−: Mild form; +: More Severe)	Relevance with Infection (P: Protective; R: Risk)
HFRS	*HLA*	Finland	HLA-B*08		PUUV	+	
			HLA-DRB1*0301		PUUV	+	
			HLA-B*27		PUUV	−	
		Slovenia	HLA-DRB1*15		PUUV	+	
			HLA-DRB1*13		PUUV & DOBV	+ (PUUV)	PUUV > DOBV
			HLA-B*35		PUUV & DOBV	+ (DOBV)	DOBV > PUUV
			HLA-B*07		PUUV		P
		China	HLA-B*46		HTNV	+	
			HLA-B*46/DRB1*09		HTNV	+	R
			HLA-B*51/DRB1*09		HTNV	+	
			HLA-DRB1*12		HTNV	−	P
	*TNF*	Finland	−308		PUUV	+	
		Belgium	−238		PUUV	+	
	*C4A*	Finland	Deletion		PUUV	+	
	*HPA-3*	China	3b		HTNV	+	R
	*PAI-1*	Finland	GG		PUUV	+	
	*Gp1A*	Finland	C		PUUV	+	
	*VE-CDH5*	Russia	*T/*T		PUUV	+	
	*GATA-3*	Finland		Higher	PUUV	+	
	*IL1-RA*	China	*/*		HTNV		R
	*IL1-1b*	China	−511		HTNV		R
	*IL1*	Finland	x		PUUV	None	
	*HPA-1*	China	x		HTNV	None	
	*CD3e*	Finland		x	PUUV	None	
	*T-BET*	Finland		x	PUUV	None	
HCPS	*HLA*	US	HLA-B*3501		SNV	+	
			HLA-DRB1*1402		SNV	+	
			HLA-B*35		ANDV	−	
		Chile	HLA-B*08		ANDV	+	
			HLA-DRB1*15		ANDV	−	
	*TNF*	Brazil	−308G/A		ARAV	+	

+ and − respectively indicate that severe or mild forms of hantavirus disease are associated with genetic variations; P and R respectively indicate that genetic variations confer protection or are associated with higher risk of hantavirus infection.

Thus, different hantaviruses seem to be processed differently through the same HLA molecules resulting in mild or severe outcomes of the disease. Studies on the genetic factors associated with disease severity due to different sympatric hantaviruses confirm this statement. In Slovenia for example, both PUUV and Dobrava virus (DOBV) are present and cause HFRS. PUUV-infected patients tend to have more frequently (32%) HLA-DRB1*13 haplotype than DOBV-infected patient (18%), especially in the severe form of PUUV disease [41]. Furthermore, DOBV-infected patients have a significantly higher prevalence of HLA-B*35 than PUUV-infected patients This allele was marginally associated with a fatal outcome of the DOBV-infected patients [41].

It is interesting to note that most of these alleles/haplotypes associated with severity of hantavirus disease are linked to abnormal immune responses or autoimmune diseases see references in [38,48]. Individuals with the haplotype HLA-B*08–HLA-DRB1*0301 are prone to normal or increased humoral immune response and a low T-cell immune responsiveness [49]. In contrast, the HLA-B*27 allele is associated with decreased production of TNF and IFN-γ by T cells [50]. These immunogenetic studies thus provided the first lines of evidence that the pathogenesis of hantavirus infection is likely to imply the immune system of the host. Further investigations are required to decipher the mechanisms linking *HLA* class I and class II gene polymorphism, T cell responses and the severity of hantavirus infection.

The tumor necrosis factor (TNF) cluster belongs to the class III region of the *HLA* complex and contains genes that encode two cytokines, TNF and LTA, and LTB, a receptor that forms heterotrimers with LTA [34]. An allele associated with high production of TNF (polymorphism at position −308) correlates with the severe clinical course of PUUV infection in Finnish patients [51] and is strongly expressed in kidneys of PUUV-infected humans [52]. *TNF* gene is partly involved in severe PUUV disease but is a less important risk factor than the HLA-B*08–HLA-DRB1*0301 haplotype [23]. In Belgium, patients with the low-producer allele of *TNF* (polymorphism at position −238) had a more severe clinical course [53,54]. In Brazil, the high-producing *TNF-a* 2 allele (−308G/A) was more frequent in HCPS patients than in individuals with antibodies but without a history of HCPS, suggesting that this allele could represent a risk factor for developing HCPS [55]. In Brazil, this *TNF-a* 2 allele association, unlike in Finland, was independent of the HLA-B*08–HLA-DRB1*0301 linkage disequilibrium. In the same study, no association was found between *TNF* alleles and the severity or case-fatality-rate of HCPS [55].

*C4A.* Deletion within the *C4A* gene encoding the C4A component of the complement system is invariably associated with the HLA-B*08–HLA-DRB1*0301 haplotype [56,57]. This is of interest since there is good evidence that complement activation contributes to the pathogenesis of PUUV infection [56]. Levels of the soluble terminal SC5b-9 complex were higher, and C3 levels were lower during the acute stage than during convalescence, especially in patients with chest x-ray abnormalities. These changes had a significant correlation with clinical and laboratory parameters of disease severity.

Polymorphism within genes encoding cytokines may modulate cytokine production during inflammation and therefore influence the outcome of hantavirus infections. Only few studies have addressed this question. Mäkelä *et al.* [58] have analyzed polymorphism of the *IL-1* family genes. They did not find any evidence of allele frequencies or genotypes affecting the clinical course of PUUV infections.

Polymorphisms of platelet glycoprotein IIb/IIIa alloatigen *(*HPA1/HPA3*)* have been investigated and HPA-3, but not HPA-1, was more frequent in Chinese patients with severe than mild HFRS [59].

In Finland, plasminogen activator inhibitor (*PAI-1*) and platelet *GP1a* were associated with severe PUUV infection [60].

Finally, the prevalence of the VE-cadherin *CDH5* genotype *T/*T was significantly higher in Russian patients with the severe form of HFRS due to PUUV than in other patients. Missense mutation c.1550T > C within the VE-cadherin gene could increase the desquamation process of endothelial cells and lead to a severe form of HFRS with complications [61].

### 2.2. Variability in Immunity-Related Gene Expression and Severity of Human Hantavirus Infections

Several associations between serum levels of cytokines TNF IL-6, IL-2, IL-8, IL-10, IFN-γ, see [62,63,64,65,66] or the intensity of platelet β3 integrin [67] and disease severity have been shown for PUUV, HTNV and DOBV infections. Genetic determinisms modulating the mRNA expression levels of the genes encoding these molecules could represent important risk factors of hantavirus disease severity. Nevertheless, only a single study has compared the levels of mRNA expression of some of these genes among patients exhibiting different progressions of hantavirus disease. Briefly, Libraty *et al.* [63] followed the mRNA expression levels of a T-cell associated gene (CD3e), a type 1 cytokine transcription factor (T-BET) and a type 2 cytokine transcription factor (GATA-3) in daily urine samples to identify risk factors for severe PUUV HFRS during acute illness (AKI). They found that only GATA-3 mRNA expression was higher in patients developing severe AKI than in those with mild AKI. They concluded that this clinical severity could be explained by excessive type 2 T-cell responses compared to type 1 T-cell responses in the kidneys. Alternatively, GATA3/Th2 response may be a negative feedback to temper immunopathology. In the near future, similar studies in other countries, for other genes and other hantavirus species, could help identifying a large array of immunogenetic factors modulating the severity of human hantavirus infections.

### 2.3. Polymorphism of Immunity-Related Genes and Human Susceptibility to Hantavirus Infections

As shown above, most of the immunogenetic studies on human hantavirus infections have looked for associations between human immunogenetics and disease severity. Only few of them investigated factors that could contribute to susceptibility to hantavirus infection. Their results have shown that all genetic variations modulating hantavirus infection risk were also involved in disease clinical severity. Hence, HLA-DRB1*09 and HLA-B*46–DRB1*09 were more common in Chinese patients with HTNV-induced HFRS than in healthy individuals [48,68]. Moreover, non-carriage of the interleukin-1 receptor antagonist (*IL-1RA*) allele 2 and the *IL-1b* (−511) allele 2 [58] as well as *HPA-3* b allele [59] were more frequent in HFRS patients than in seronegative controls. These alleles/haplotypes could thus be identified as genetic risk factors associated with the susceptibility to hantavirus infections [59].

In turn, HLA-B*07 and HLA-DRB1*12 could have a protective role, respectively, against PUUV infection in Slovenia [69] and HTNV infection in China [68].

## 3. Impact of Immunity-Related Genes on the Risk of Hantavirus Infection in Rodents

### 3.1. Kinetics of Immunity-Related Gene Expression During Hantavirus Infection in Rodents

The kinetics of immunity-related gene expression has been analyzed during experimental hantavirus infection for several rodent/hantavirus models. As the course of infection may differ among individuals [32,33,70], comparing these dynamics turned out to be relevant to the identification of immunogenetic variations underlying these differences. For now, two main questions have been investigated and are summarized below: do variations in immunity-related gene expression mediate sex differences in hantavirus infections? Do they explain the persistence or the clearance of hantaviruses in rodent reservoirs?

#### 3.1.1. Immunity-Related Gene Expression and Sex Differences in Hantavirus Infections

Longitudinal studies in reservoirs of hantaviruses have highlighted that in wild rodent populations, more males than females are infected in mature animals only, but not in subadult, *i.e.*, non-breeding ones e.g., [71,72,73,74]. Sex-based differences in gene expression could modulate these patterns in mature rodents. Klein *et al.* [75] revealed that about 1800 genes with known function were differentially expressed between sexually mature male and female Norway rats (*Rattus norvegicus*) after experimental Seoul (SEOV) infections. Up to 180 were immunity-related genes that showed a pattern of up-regulation into the lungs of females compared to males. Associated functions included inflammatory (e.g., TNF-α, TNF-αR, IL-1R, IL-1RAcP) and antiviral (eIF-2α, IFN-γR, STAT-6, (IRF)-1) responses as well as MHC, Ig and T cell marker proteins [75,76]. In addition, gene expression of heat shock proteins was higher in SEOV infected males than in females, indicating a more elevated cellular stress [75]. These studies therefore suggest that both differences in innate and acquired immunity-related gene expression could mediate dimorphic responses in rodent reservoirs to hantavirus infections.

#### 3.1.2. Immunity-Related Gene Expression and Persistence/Clearance of Hantavirus Infections

Hantaviruses often cause an acute infection followed by a persistent phase in reservoir rodents. However, variable patterns of infection have been observed among infected individuals, even within a given reservoir species. For example, Botten *et al.* [77] revealed two distinct patterns of infection (based on the levels and the distribution of viral RNA) during the persistent phase of SNV infection in deer mice. Some individuals exhibited a “restricted” pattern of viral replication and antigen expression (antigen expression was for example detected in fewer than three of the tissues examined), while others showed a “disseminated” pattern of infection (antigen expression was observed in five to nine of the tissues examined). Studying the kinetics of rodent gene expression following hantavirus infection has helped understanding these phenomena. In particular, it allowed discriminating several mechanisms explaining hantavirus persistence, including immune evasion, direct suppression or modification of host immune responses.

The comparison of cytokine gene expression profiles between T cell lines in acutely and persistently infected deer mice revealed an increase of *TGF-β* and *FoxP3* mRNA expression and a decrease of *IL-10* and *IL-4* expression during the persistent phase of SNV infection in most of the lines studied [78]. Similar results were obtained by Easterbrook *et al.* [79,80] based on the study of the persistent phase of SEOV infection in male Norway rats. Increased levels of *FoxP3* and *TGF-β* mRNA expression were observed in the lungs of SEOV infected rats compared to uninfected ones. In contrast, the levels of *IL-10*, *IL-1b*, *IL-6* and *TNF* gene expression were reduced. Easterbrook *et al.* [80] also showed using SEOV infected male Norway rats that proinflammatory responses were elevated (e.g., high expression of *IL-6*, *CCL2 and CCL5* genes) and that regulatory responses (e.g., expression of *TGF-β* and *Fox-P3*) were not induced in spleens, an immunity-related organ where hantavirus replication is low. Similar results were observed following immunity-related gene expression in spleen of deer mice infected with ANDV [33], although responses were more heterogeneous among individuals. This was probably due to the fact that rodents were more inbred in this experiment than in the one described above. These common modifications of immunity-related gene expression during the persistent phase seemed to depend on a concomitant increase of regulator T cells. By suppressing proinflammatory responses, they could on one hand contribute to the tolerance of reservoirs to hantavirus infection and their associated pathogenesis, and on the other hand, lead to hantavirus persistence in the host.

### 3.2. Immunogenetics and Rodent Susceptibility to Hantavirus Infections

#### 3.2.1. Sequence Polymorphism of Immunity-Related Genes between Reservoir and Non-Reservoir Species and Their Association with Susceptibility to Hantavirus Infection

Rodent species exhibit different capacities as reservoirs of hantaviruses. For example, hantavirus infections are supposed to be asymptomatic and chronic in their rodent reservoirs, however some rodent species are known to be non-reservoirs of hantaviruses. The Syrian hamsters (*Mesocricetus auratus*) and the house mice for example do not carry any hantavirus in the wild and are known to mimic human pathogenesis or to die when being infected respectively with ANDV and HTNV see references in [81]. The fact that phylogenetically related rodent species share similar properties allowing a given hantavirus to replicate compared to distant related ones [82] is a first argument indicating that genetics might modulate these variations. Inter-specific molecular differences in the genes encoding proteins involved in virus entry into host cells are good candidates to test this hypothesis see for example [5].

To date, no receptor for hantaviruses has been defined nor suggested in animal host species. Our current knowledge is based on *in vitro* or *in vivo* analyses conducted on laboratory rodent species that are not reservoirs of hantaviruses. Therefore, mechanisms of viral entry in reservoir animals remain unknown. Nevertheless, it could be interesting to analyse the polymorphism and the phylogenies of the genes encoding these receptors or other proteins. Several candidate genes can be identified from the literature. The gene fragment encoding the plexin–semaphorin–integrin (PSI) domain of the α_v_β_3_ integrin is involved in viral attachment for several pathogenic hantaviruses in humans [83,84,85,86,87]. Single amino acid changes performed through mutagenesis were shown to modify hantavirus recognition and subsequent infection of culture cells. Raymond *et al.* [86], then Matthys *et al.* [88] showed that mutagenizing the murine PSI domains to homologous human residues (substituting serine for a proline—S32P or asparagine to aspartic acid—N39D respectively) allowed these mutant polypetides to inhibit hantavirus infection (NY-1V and ANDV respectively). Among other potential candidates, the β1 integrin, the complement decay-accelerating factor (DAF) and GC1QR (also known as C1QBP) should be investigated because studies have emphasized their potential role in hantavirus entry into cells (Vero and human cells) [84,89,90]. Further phylogenetic analyses of the genes encoding these receptors could provide new information on their potential implication in hantavirus entry in reservoir cells.

#### 3.2.2. Sequence/Expression Variability of Immunity-Related Genes between Rodent Populations Sampled in Endemic and Non-Endemic Areas and Their Associations with Susceptibility to Hantavirus Infection

Several works based on experimental infections of rodents have revealed that hantavirus infectivity varies among individuals of a same species [31,32,33,77,91]. Although infection is most of the time asymptomatic, changes in tissue morphology similar to those associated with SNV infections in humans (pulmonary oedema, periportal hepatitis) have been reported once in white-footed mouse, *Peromyscus leucopus*, experimentally infected with New-York virus [92]. Similar histopathologies were observed in wild caught deer mice, *Peromyscus maniculatus*, infected with SNV [93]. Strong correlations were observed between the detection of pulmonary histopathological findings and the amount of viral antigen detected in organs, suggesting that these morphological changes were caused by SNV infection. These results have nevertheless to be taken carefully as such evidence of lesions remain rare compared to the large number of experimentally infected rodents which did not show any sign of pathology. Kallio *et al.* [31] exposed naive bank voles, *Myodes glareolus,* to beddings previously contaminated by PUUV. They showed that infection outcomes were highly variable among recipient voles, independently of sex or age. Guivier *et al.* [94] evaluated whether immunity-related gene polymorphism could explain these differences. Unfortunately, no significant associations could be detected between infection success and immunity-related gene polymorphism of these bank voles. *Dqa Mhc* class II gene was monoallelic among the 101 bank voles analyzed from this experimental dataset. The relative risk associated with Mygl-Drb*117 was high (RR = 4.82, *p* = 0.062) although not significant. This absence of relationship was likely to be explained by the loss of genetic variability that occurred during the long-term multigenerational captivity of these rodents [94].

Other studies investigated the influence of immunogenetic background on hantavirus risk in rodents, using natural populations sampled in endemic and non-endemic areas for hantaviruses. To our knowledge, such studies have only been conducted on bank voles, the reservoir of PUUV. Patterns of spatial genetic differentiation have been contrasted between presumed neutral markers and immunity related genes. Comparing patterns of population genetic differentiation observed for these types of genes allows for detecting signatures of contemporary selective processes [95]. Associations between immunity-related gene polymorphism and PUUV infectious status (infected/non-infected) are next conducted to infer whether such selection might be driven by *M. glareolus*/PUUV interactions. These studies are summarized below.

##### 3.2.2.1. *Mhc* Class II Genes: *Drb*, *Dqa*

Studying associations between *Mhc* haplotypes and hantavirus infections in wild rodents appeared an obvious aim in light of the human medical literature previously cited. Studies focused on the class II *Drb* and *Dqa* genes because of their high levels of polymorphism. Class I genes are more difficult to examine because of the large number of duplicated copies, which prevents the amplification of all alleles and blur the assessment of genotypes.

**Figure 1:**
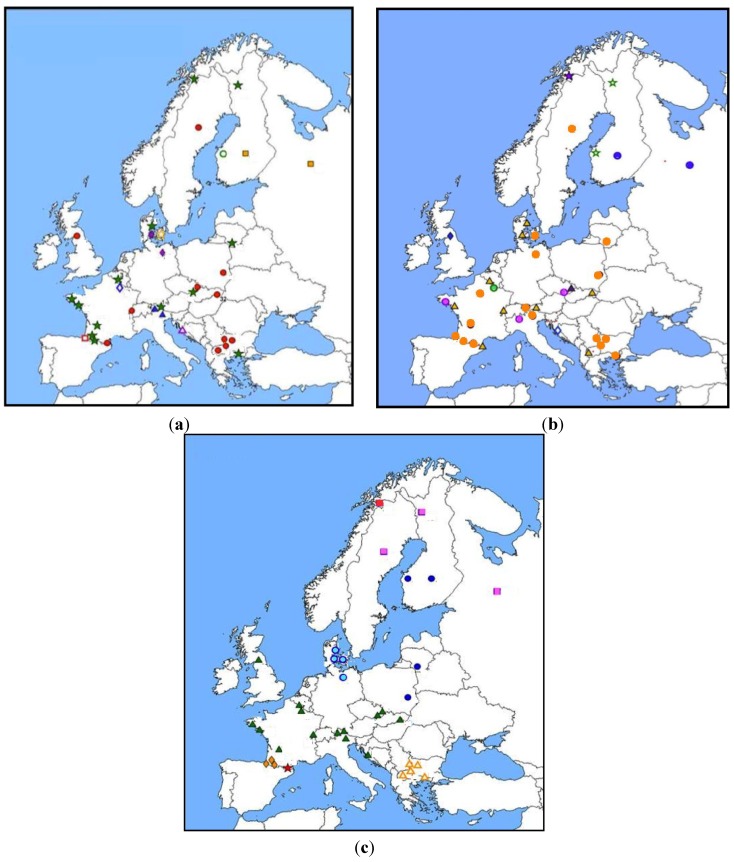
Distribution of *Mhc* (**a**) *Dqa* and (**b**) *Drb* exon2; and (**c**) mitochondrial cytochrome *b* (*cyt b*) polymorphism in *M. glareolus* populations over Europe (from [95,98]). Spatial clustering was defined using Spatial Analysis of MOlecular VAriance (SAMOVA). Populations belonging to a same cluster are represented by a same symbol.

About 350 bank voles coming from 38 European localities have been genotyped for the cytochrome *b* (mitochondrial marker, supposed to evolve neutrally), *Dqa* and *Drb* class II genes [96,97]. Haplotype distributions were analyzed and compared between genes to study the relative influence of history (bottleneck, migration, expansion) and natural selection forces (including PUUV mediated selection) acting on *Mhc* genes. A spatial analysis of molecular variance (SAMOVA) was applied to find population clusters that maximize molecular variance among population groups [98]. Clusters were defined independently for each gene. High levels of incongruence were observed, both between *Mhc* and mitochondrial genes and between *Dqa* and *Drb* genes Figure 1 [99]. For example, the 10 clusters found based on *Drb* haplotype distribution did not correspond neither to the phylogeographic groups expected from the European colonization/recolonization history of the bank voles (assessed via cytochrome *b* analysis) [100], nor to PUUV (presence/absence of the virus or distribution of the different lineages previously described by Nemirov *et al.* [101]). These results suggested that selection is likely to influence the evolution of the *Drb Mhc* class II gene, and that multifactorial pressures (including other pathogens for example) rather than PUUV risk alone mediate this selection. The distribution of *Drb Mhc* class II gene polymorphism should therefore not be simply used to infer the chance of PUUV infection in bank voles.

Genotype-phenotype associations were examined in large European datasets (about 200 bank voles, 65 PUUV-seropositive ones) that included bank voles sampled in Fennoscandia (Finland and Sweden), and in French and Belgian (the Ardennes and the Jura) PUUV-endemic areas. *Dqa* gene polymorphism did not influence the probability of bank voles being infected with PUUV in most of geographic localities considered [94]. However, in the French Jura (corresponding to a recently identified area of PUUV endemicity), significant negative associations were detected between the presence of anti-PUUV antibodies and both Cgl-DQA-05 and Cgl-DQA-12 alleles (RR = 0.21 and 0.57, respectively). In a lesser extent, Cgl-DQA-09 and Cgl-DQA-11 were more present in PUUV infected voles (positiveassociation, RR = 2.83 and 2.07 [102]). These results have nevertheless to be taken cautiously as only nine bank voles among the 98 studied were PUUV seropositive in this sampling.

*Drb* haplotypes significantly discriminated seropositive bank voles from seronegative ones, but only in the Fennoscandian localities. The allele Mygl-Drb*03 exhibited a high relative risk (RR) in Finland (Ilmajoki, RR = 3.73). The allele Mygl-Drb*93 was associated with high RR in Sweden (Västerbotten, RR = 2.95). None of these alleles were found in the Ardennes [94]. None of the other alleles detected in the French and Belgian Ardennes were associated with the probability for a rodent to be PUUV infected. A recent population genetic analysis comparing the patterns observed for neutral microsatellites and the *Drb* gene in localities sampled in PUUV endemic and non-endemic areas Northand South French Ardennes, see [103] did not reveal any signatures of selection at this *Mhc* class II gene [97].

Altogether, these results mirror in some extents what was previously described in humans. Associations between the presence of particular *Mhc* haplotypes and PUUV antibodies were detected and showed a high level of geographic variability, probably reflecting local adaptations between hosts and viruses. These adaptations seem stronger in Fennoscandian localities (detection of associations and signatures of selection), probably because the levels of PUUV prevalence are higher and possibly because co-adaptation between *M. glareolus* and PUUV has a longer history. Whether these *Mhc* haplotypes directly confer a higher susceptibility or PUUV resistance in bank voles (or in other rodent species) can not be determined based on these field studies solely.

##### 3.2.2.2. *Tnf*

Guivier *et al.* [16,104] have analyzed the distribution of *Tnf* promoter polymorphism in *M. glareolus* populations over Europe (Sweden, Finland, Germany, France, and Czech Republic). They hypothesized that spatial genetic differentiation between endemic and non-endemic areas could be mediated by PUUV and that polymorphism should reflect variation in *Tnf* gene expression. Sixteen single nucleotide polymorphisms (SNPs) were detected, among which three exhibited frequencies that allowed performing further statistical analyses (SNP −390 C/T, −296 G/A et −302 GG/~~). Two of them showed interesting patterns with regard to the variation of allelic frequencies between localities. Genetic differentiation indices between France and Czech Republic or between all pairs including the Finnish locality were significant. The allele −296G and the genotype −302~~ (~meaning deletion) were observed at low frequencies in France, Czech Republic and Slovakia but were highly represented in Finland where PUUV is highly prevalent.

Further analyses of associations between these SNPs and both *Tnf* splenic expression or PUUV infection suggested that TNF response could also be important in *M. glareolus*/PUUV interactions. The relative risk RR, see [105] of PUUV infection associated with these SNPs varied between 0.93 (Finland) and 2.82 (French Ardennes), indicating that voles carrying rare alleles (−296 G or −302~~) were at least twice more likely to be infected by PUUV than voles exhibiting common alleles [104]. Rohfritsch *et al.* [97] carried out a population genetic analysis to look for selection acting on the *Tnf* promoter between the endemic and non-endemic localities of the French Ardennes (North-South transect). They revealed a higher genetic differentiation at site −296 than expected under the neutral assumption, especially when comparing northern (endemic) and southern (very low PUUV seroprevalence) localities. Therefore, population genetics analyses have revealed that the distribution of particular *Tnf* promoter SNPs between bank vole populations could not be explained by neutral evolutionary forces only. On the other hand, associations were detected between these SNPs and the risk of PUUV infection in bank voles. Selection acting on *Tnf* promoter could therefore be linked to PUUV, either indirectly, or potentially directly. Indeed, several ongoing studies are providing evidence of negative effects of PUUV on different components of vole fitness, including survival [28,29,71]*.*

**Figure 2:**
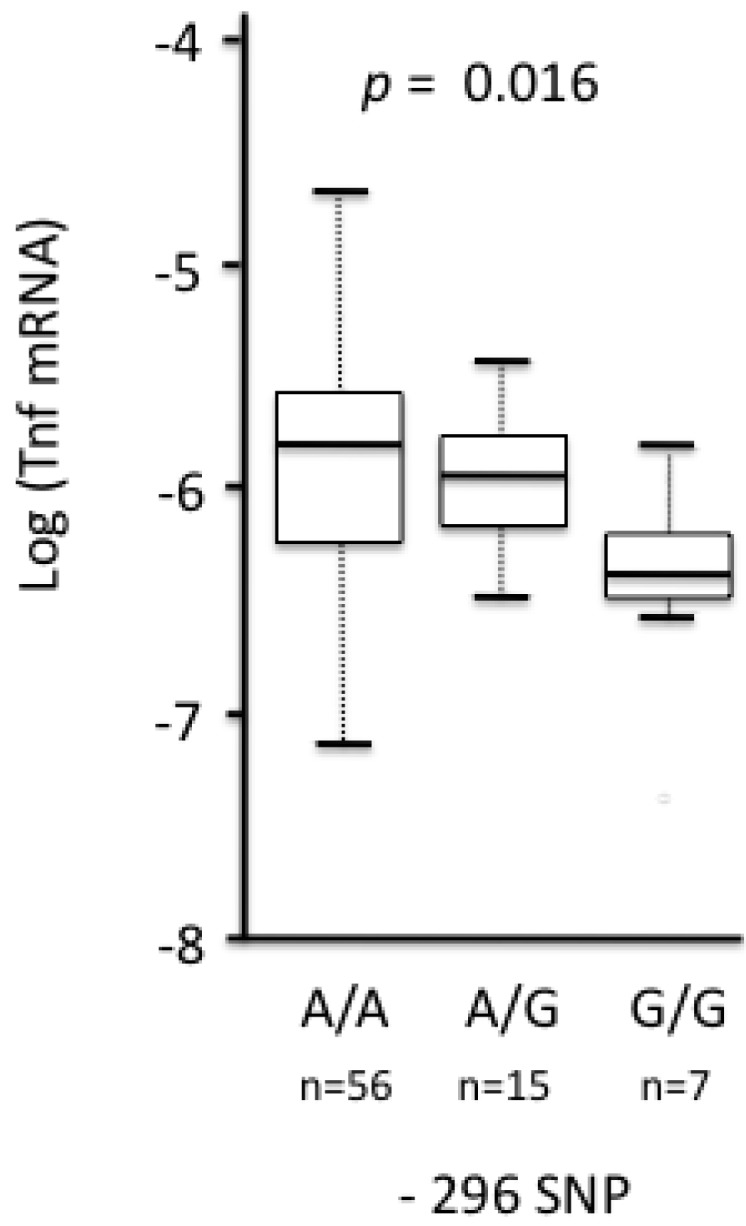
Relationship between variation at position −296 of the *Tnf* promoter and the log-transformed relative expression of *Tnf* (here: log (*Tnf* mRNA)) in European bank voles. Relative expression was estimated as *[(E_Tar_ + 1)^CpTar^]/[(E_Ref_ + 1)^CpRef^] with E_Tar_, E_Ref_, Cp_Tar_ and Cp_Ref_ being, respectively, the average efficiencies of the target (Tnf) and reference (β-actin) genes and the crossing points of the target and reference genes* (see [104]). ANOVA was first performed and emphasized significant differences of *Tnf* relative expression among *Tnf* promoter genotypes (ANOVA, F_2,75_ = 4.002, *p* = 0.022). Further Tukey-Kramer tests showed that voles with genotype -296 A/A exhibited a significantly higher relative expression of *Tnf* than those with −296 G/G genotype (Tukey–Kramer test, *p* = 0.016). Boxes represent the first and third quartiles of the distribution. Horizontal black lines correspond to medians. The vertical dashed lines correspond to 1.5 times the interquartile range.

In Europe, polymorphism at site −296 was significantly associated with the relative expression of *Tnf* gene detected in the spleen [104]. Homozygotes −296 A/A had higher mRNA levels of *Tnf* gene than the −296 G/G heterozygotes (Figure 2).

**Figure 3:**
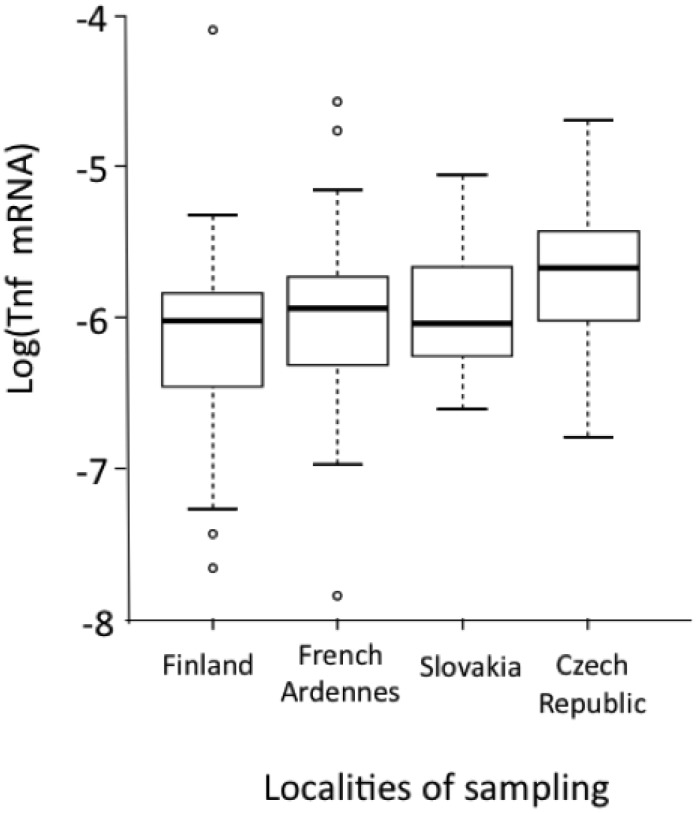
Geographic variations of the levels of *Tnf* relative expression (see above for detailed formula) detected in bank voles from four European localities [16]. A multiple linear regression with selection model procedure was performed; locality was the only significant effect detected (*F_3,132_* = 5.204; *p* = 0.002). Boxes represent the first and third quartiles of the distribution. Horizontal black lines correspond to medians. The vertical dashed lines correspond to 1.5 times the interquartile range. The circles represent the values superior and inferior to 1.5 times the interquartile range.

mRNA levels were next compared between localities (Figure 3a). They were significantly lower in bank voles sampled in PUUV endemic areas (Finland, French Ardennes) than in a locality where PUUV has not yet been recorded (Eastern part of Czech Republic). Using the North-South transect in the Ardennes, Guivier *et al.* [16] also revealed that higher levels of *Tnf* relative expression were associated with lower PUUV loads in wild bank voles [104].

TNF plays an important role in immune responses but also induces pathophysiologic changes. It has therefore been suggested that it could mediate two types of responses against PUUV: resistance when highly produced and tolerance when weakly produced. The latter corresponds to an adaptive process limiting pathogeneses at the expense of pathogen growth or reproduction [104,106]. Altogether, the studies described above have shown that higher levels of *Tnf* gene expression could limit PUUV infection in bank voles. They also evidence that some geographic variability in the level of *Tnf* gene expression, genetically determined by *Tnf* promoter polymorphism, could reflect the co-adaptation history between PUUV and bank voles. In endemic areas with continuously high PUUV prevalence (e.g., Finland, French Northern Ardennes), strong co-adaptation histories between *M. glareolus* and PUUV could have selected for higher levels of tolerance to PUUV infections (*i.e*., lower levels of TNF production) than in populations where prevalence levels of PUUV are low (Czech Republic, French Southern Ardennes).

##### 3.2.2.3. Other Genes (*Tlr4*, *Tlr7*, *Mx2*, *β3 Integrin*)

Coding sequence polymorphism and gene expression of few other candidate genes have also been compared using the French Ardennes design (North-South transect). Sequence polymorphism of the genes encoding TLR4 (exon3), TLR7 (exon3), Mx2 (exons 5 to 14) and the PSI domain of β3 integrin has been analyzed for about 300 bank voles sampled in the French Ardennes. Respectively 10, 1, 4 and no SNPs were detected in these individuals. Population genetic analyses did not reveal any signature of selection acting on these genes when comparing endemic (Northern) and non-endemic (Southern) localities [96].

Like other Mx proteins, Mx2 is an interferon-induced gene product involved in antiviral response. Because Mx2 is known to limit PUUV replication in humans and in cell cultures [106], and to induce pathological symptoms when overproduced [108,109], Guivier *et al.* [16] analysed the variability of *Mx2* gene expression between areas of high and low PUUV seroprevalence in the French Ardennes. Similar results as those observed with *Tnf* were described. The mRNA levels of *Mx2* genes were negatively correlated with PUUV loads in infected bank voles, corroborating the idea that Mx2 might limit PUUV replication in bank voles. They also showed that bank voles from forests (higher PUUV seroprevalence) had lower levels of *Mx2* gene expression than voles living in fragmented hedge networks lower PUUV seroprevalence, higher genetic drift, see [110]. Again, these lower levels of expression may be considered to reflect tolerance mechanism against pathogens (including PUUV).

Applying immunogenetics to the study of *M. glareolus*/PUUV interaction in natural populations has provided new arguments in favour of tolerance mechanisms evolving in bank voles, directly or indirectly mediated by PUUV. Histories of host-pathogen co-adaptation, differences in biotic and abiotic environmental conditions are factors that could maintain and shape variation in the amplitude of tolerance among bank vole populations. These conclusions are derived from correlative patterns based on the study of few immunity-related genes. Genomic approaches or experimental approaches will help confirm these results and detail the mechanisms underlying tolerance to PUUV and its variation over Europe. In future, similar studies should be conducted on other rodent/hantavirus models to test if this phenomenon may be generalized at a larger evolutionary scale.

## 4. Discussion: The Evolutionary Perspectives

### 4.1. Geographic Distribution of Susceptible Haplotypes and the Risk of Hantavirus Emergence

As detailed above, the analyses of genetic variation modulating the susceptibility, hantavirus replication or severity of disease, have usually been performed at the population level (one locality or one country) in reservoirs or in humans. Congruent or incongruent patterns may exist when results are compared between populations. Nevertheless, no or few lessons have been learned from these comparisons but see in rodents, [16,104]. To our knowledge, there is no real wish to conduct a worldwide human case study. Until now, research focused on a single country or on a specific hantavirus. Including a wide range of populations concerned with one or several specific hantaviruses could provide more generic conclusions on the role of immunogenetics on human susceptibility to hantaviruses.

Furthermore, such research would have important consequences in terms of public health. In humans, allele frequencies are now available for several millions of single nucleotide polymorphisms (SNPs) in worldwide populations [111,112]. These data have been used to evaluate genome-wide association studies for many diseases 250 studies referenced in 2012, see [18]. It would be worth exploiting these genomic data to evaluate the genetic basis of human susceptibility to hantavirus infection at a large genomic scale, and to explore potential geographic variations (e.g., between the New and Old Worlds, or between European and Asian countries). Ultimately, it could be envisaged to map genetic risks of HFRS across Europe and Asia or HCP in the Americas. These distribution maps could help targeting regions where HFRS risk is high but still underestimated or unrecognized. Such regions would be the first priority for informing health professionals and developing communication strategies to the general public.

Comparison of the geographic distribution of allele frequencies for “neutral” SNPs and SNPs that are associated with susceptibility to hantaviruses or severity of hantaviral disease may highlight the historical and selective evolutionary forces acting on these candidate SNPs and shaping the observed distribution of polymorphism. Several studies have been conducted in this framework for different immunity-related genes. Barreiro *et al.* [113] considered two innate immunity-related genes sharing very close physical vicinity (Cd209 and Cd209l). They emphasized that history and selection had lead to different outcomes of innate immunity-related gene polymorphism. Traces of ancient population structure in Africa and strong functional constraints explained the diversity of Cd209 gene while balancing selection was likely to generate the high level of diversity observed at the Cd209l gene. A major difficulty may reside in the identification of the selection pressure acting on candidate genes and shaping the distribution of their polymorphism at large geographical scales see for example in mammals [96]. Nevertheless, some significant examples exist, including the comparative evolutionary histories of thalassemia and malaria [114]. The application of concepts of evolutionary biology to the study of immunogenetic factors affecting susceptibility to hantaviruses or clinical course of hantaviral disease in humans should be promising approaches in the near future.

### 4.2. Evolution of Tolerance in Rodents and Its Epidemiological Consequences

Several studies have emphasized the high level of variability in reservoir susceptibility to a given hantavirus or in their ability to limit hantavirus replication [16,104,115]. These results may be interpreted in terms of tolerance to hantavirus as some of the molecules involved in this process can lead to immunopathologies when over-produced (e.g., TNF-α, Mx2). In turn, this individual variability of tolerance can strongly impact hantavirus epidemiology. Individuals exhibiting high levels of tolerance will play a major role in hantavirus persistence, dissemination and transmission dynamics. Tolerance has neutral or positive effects on pathogen fitness [106,116]. Tolerant individuals may hence support higher viral loads, leading to higher quantities of viral particles potentially excreted into the environment. Such individuals might therefore be considered as super-spreaders of hantavirus [117]. It appears important to identify these super-spreaders within and between reservoir populations/species. This would improve our knowledge about hantavirus eco-epidemiology, and ultimately would allow to adapt public health prevention strategies. It is therefore crucial to evaluate the veracity of reservoir tolerance to hantaviruses and super-spreading events, to identify its genetic bases as well as its phenotypic plasticity. In particular, the role of co-infection has been emphasized to explain some cases of super-spreading see examples in [118]. How and which additional infections modulate the quantity of hantavirus particles excreted in the environment, and how reservoir immunogenetics might influence the probability of such co-infections through antagonistic pleiotropy (*i.e*., genetic trade-offs) are important questions to address in future research.

### 4.3. Difficulties to Define What Is a Non-Reservoir Species for Hantaviruses

The notion of non-reservoir species is difficult to define because the absence of evidence (*i.e*., detection of a virus) is not evidence in itself.

Non-reservoir species may correspond to species that have never been exposed to the virus or species that have never been conceived as potential reservoirs (because of their taxonomic rank or because of the regions they inhabit). Until recently, hantaviruses were thought to be maintained in nature in rodent reservoirs from Eurasia and Americas solely. However, new hantavirus species have been described in African rodents e.g., Sangassou, [119] but also in small mammals other than rodents e.g., Talpidae, Soricidae, Chiroptera, see for a recent review [20]. Non-reservoir species may also be those that die extremely quickly upon infection hence limiting the probability of detecting positive individuals in wild populations. Finally, non-reservoir species may also concern those in which hantavirus could not be able to enter and/or replicate within cells. In these two latter cases, no hantavirus would be detected and the term “non-reservoir” would embrace species highly susceptible and highly resistant to the virus.

A last but not least difficulty arises from the fact that different hantaviruses may produce opposite effects according to the host species considered. For example, Maporal virus (MAPV), a hantavirus that was originally isolated from an arboreal rice rat, *Oecomys bicolor*, causes disease in the Syrian golden hamster, *Mesocricetus auratus*, that is clinically and pathologically remarkably similar to HCPS [120]. Note that there is presently no evidence that MAPV is pathogenic in humans. After infection with ANDV, hamsters also develop HCPS-like disease that faithfully mimics the human condition with respect to incubation period and pathophysiology of disease. On the contrary, the closely related human pathogen SNV can replicate in hamsters but does not cause overt disease while Old World hantaviruses such as PUUV, HTNV, SEOV, and DOBV only produce subclinical infections [121]. Thus disease and infection outcomes do not seem to correlate in this animal model with human disease-causing potential.

Until now, rodent models such as the golden hamster or the laboratory mouse are considered as useful ones to study the pathogenesis of hantavirus disease in humans and to assess the role of potential therapeutic agents. In parallel, it would be worth comparing immunology in reservoir and non-reservoir species for which rodent host/hantavirus interactions lead to radically different outcomes. It could help emphasizing mechanisms and genetic characteristics underlying such differences. In particular, unraveling the processes governing persistent infection and clearance of the virus in the natural hosts could open new avenues for human medical research.

### 4.4. Differences in Hantavirus Virulence

This review deliberately focused on rodent reservoir and human immunogenetics. Comparative genomics of hantaviruses is an obligatory counterpart to fully understand reservoir or human/hantavirus interactions and co-adaptation. Such approach coupling hantavirus sequencing from infected wild animals and humans could help to solve some of the unresolved questions concerning hantaviruses, including the determinants of pathogenicity or host switching, the receptor for entry into reservoir cells, *etc.*

Genetically and antigenically closely related hantaviruses can show large differences in virulence. Recently a subdivision of the DBV into four closely related genotypes was proposed [119]—Dobrava, Sochi, Kurkino, and Saaremaa. These genotypes correspond to different phylogenetic lineages, and display specific host reservoirs, geographical distribution, and pathogenicity for suckling mice and humans. More detailed studies of these closely related hantavirus genotypes, causing either life-threatening (Dobrava, Sochi), relatively mild infection (Kurkino) or possibly only subclinical human infections (Saaremaa), could reveal the genetic determinants of virus-host interaction mechanisms leading to virulence.

In addition, *in vitro* hantavirus infections of cultured cells (e.g., Vero E6, CHO, HUVECs) have suggested that non-pathogenic hantaviruses use β_1_ integrin as receptor for cell entry while the pathogenic ones use β_3_ integrin [123]. Hantaviruses carried by the *Microtus*-voles, such as Tula virus (TULV), or Sangassou, which is harbored by the African wood mouse, *Hylomyscus simus*, were demonstrated to infect humans, although this seems to be rare [124,125,126], but to use β_1_ integrin, at least in cell culture models. This highlights the needs to better understand the receptors used by pathogenic or non-pathogenic hantaviruses and the potential links between these receptors and hantavirus pathogenicity in humans. Whether the newly found shrew-, mole- and bat-borne viruses infect other animals including humans, and if so with which consequences, also remains to be elucidated. By this way, hantavirus comparative genomics would help to reveal some of the genetic determinants of human pathogenicity.

## 5. Concluding Remarks

In summary, this review aimed at highlighting a number of important immunity-related genes that seem to be associated with the clinical course of hantaviral disease in humans, and the susceptibility of humans and rodents to hantaviruses. Beyond this list, we wanted to emphasize the necessity, in the very near future, to “infect” the classical human immunogenetics approach both with evolutionary biology and with the datasets produced by the human genome projects. This combination of approaches, the future accumulation of genetic data using new generation sequencing technologies and genome-wide association studies, as well as closer collaborations between researches developed on wild reservoirs and humans, should ultimately improve our knowledge of hantavirus risk and epidemiology.

## References

[1] Chapman S.J., Hill A.V.S. (2012). Human genetic susceptibility to infectious disease. Nat. Rev. Genet..

[2] Geraghty D.E., Daza R., Williams L.M., Vu Q., Ishitani A. (2002). Genetics of the immune response: Identifying immune variation within the mhc and throughout the genome. Immunol. Rev..

[3] Cooke G.S., Hill A.V.S. (2001). Genetics of susceptibility to human infectious disease. Nat. Rev. Genet..

[4] Trowsdale J., Knight J.C. (2013). Major histocompatibility complex genomics and human disease. Genom. Hum. Genet..

[5] Do Valle T.Z., Billecocq A., Guillemot L., Alberts R., Gommet C., Geffers R., Calabrese K., Schughart K., Bouloy M., Montagutelli X. (2010). A new mouse model reveals a critical role for host innate immunity in resistance to rift valley fever. J. Immunol..

[6] Finlay E.K., Berry D.P., Wickham B., Gormley E.P., Bradley D.G. (2012). A genome wide association scan of bovine tuberculosis susceptibility in holstein-friesian dairy cattle. PLoS One.

[7] Jones K.E., Patel N.G., Levy M.A., Storeygard A., Balk D., Gittleman J.L., Daszak P. (2008). Global trends in emerging infectious diseases. Nature.

[8] Acevedo-Whitehouse K., Cunningham A.A. (2006). Is mhc enough for understanding wildlife immunogenetics?. Trends Ecol. Evol..

[9] Tschirren B., Andersson M., Scherman K., Westerdahl H., Raberg L. (2012). Contrasting patterns of diversity and population differentiation at the innate immunity gene toll-like receptor 2 (TLR2) in two sympatric rodent species. Evolution.

[10] Turner A.K., Begon M., Jackson J.A., Paterson S. (2012). Evidence for selection at cytokine loci in a natural population of field voles (*Microtus agrestis*). Mol. Ecol..

[11] Tollenaere C., Duplantier J.M., Rahalison L., Ranjalahy M., Brouat C. (2011). Aflp genome scan in the black rat (*Rattus rattus*) from Madagascar: Detecting genetic markers undergoing plague-mediated selection. Mol. Ecol..

[12] Bonneaud C., Balenger S.L., Zhang J., Edwards S.V., Hill G.E. (2012). Innate immunity and the evolution of resistance to an emerging infectious disease in a wild bird. Mol. Ecol..

[13] Jensen L.F., Hansen M.M., Mensberg K.L., Loeschcke V. (2008). Spatially and temporally fluctuating selection at non-mhc immune genes: Evidence from tap polymorphism in populations of brown trout (*Salmo trutta*, l.). Heredity.

[14] Tschirren B., Andersson M., Scherman K., Westerdahl H., Mittl P.R.E., Raberg L. (2013). Polymorphisms at the innate immune receptor TLR2 are associated with borrelia infection in a wild rodent population. Proc. Roy. Soc. Lond. B.

[15] Bradbury J. (2004). Ancient footsteps in our genes: Evolution and human disease. Gene variants selected during evolution may underlie many common diseases. Lancet.

[16] Guivier E., Galan M., Henttonen H., Cosson J.F., Charbonnel N. (2014). Landscape features and helminth co-infection shape bank vole immunoheterogeneity, with consequences for Puumala virus epidemiology. Heredity.

[17] Tollenaere C., Bryja J., Galan M., Cadet P., Deter J., Chaval Y., Berthier K., Ribas Salvador A., Voutilainen L., Laakkonen J. (2008). Multiple parasites mediate balancing selection at mhc class ii genes: Insights from multivariate analyses and population genetics in the fossorial water vole. J. Evol. Biol..

[18] Vasseur E., Quintana-Murci L. (2013). The impact of natural selection on health and disease: Uses of the population genetics approach in humans. Evol. Appl..

[19] Jonsson C.B., Figueiredo L.T., Vapalahti O. (2010). A global perspective on hantavirus ecology, epidemiology, and disease. Clin. Microbiol. Rev..

[20] Vaheri A., Strandin T., Hepojoki J., Sironen T., Henttonen H., Mäkelä S., Mustonen J. (2013). Uncovering the mysteries of hantavirus infections. Nat. Rev. Microbiol..

[21] Schmaljohn C., Hjelle B. (1997). Hantaviruses: A global disease problem. Emerg. Infect. Dis..

[22] Vaheri A., Henttonen H., Voutilainen L., Mustonen J., Sironen T., Vapalahti O. (2013). Hantavirus infections in Europe and their impact on public health. Rev. Med. Virol..

[23] Mäkelä S., Mustonen J., Ala-Houhala I., Hurme M., Partanen J., Vapalahti O., Vaheri A., Pasternack A. (2002). Human leukocyte antigen-B8-DR3 is a more important risk factor for severe Puumala hantavirus infection than the tumor necrosis factor-alpha(-308) g/a polymorphism. J. Infect. Dis..

[24] Lundkvist A., Plyusnin A., Leitner T. (2002). Molecular epidemiology of hantavirus infections. The Molecular Epidemiology of Human Viruses.

[25] Makary P., Kanerva M., Ollgren J., Virtanen M.J., Vapalahti O., Lyytikäinen O. (2010). Disease burden of Puumala virus infections, 1995–2008. Epidemiol. Infect..

[26] Childs J.E., Glass G.E., Korch G.W., LeDuc J.W. (1989). Effects of hantaviral infection on survival, growth and fertility in wild rat (*Rattus norvegicus*) populations of Baltimore, Maryland. J. Wildl. Dis..

[27] Meyer B.J., Schmaljohn C.S. (2000). Persistent hantavirus infections: Characteristics and mechanisms. Trends Microbiol..

[28] Kallio E.R., Voutilainen L., Vapalahti O., Vaheri A., Henttonen H., Koskela E., Mappes T. (2007). Endemic hantavirus infection impairs the winter survival of its rodent host. Ecology.

[29] Tersago K., Crespin L., Verhagen R., Leirs H. (2012). Impact of Puumala virus infection on maturation and survival in bank voles: A capture-mark-recapture analysis. J. Wildl. Dis..

[30] Luis A.D., Douglass R.J., Hudson P.J., Mills J.N., Björnstad O.N. (2012). Sin Nombre hantavirus decreases survival of male deer mice. Oecologia.

[31] Kallio E.R., Klingström J., Gustafsson E., Manni T., Vaheri A., Henttonen H., Vapalahti O., Lundkvist A. (2006). Prolonged survival of Puumala hantavirus outside the host: Evidence for indirect transmission via the environment. J. Gen. Virol..

[32] Hardestam J., Karlsson M., Falk K.I., Olsson G., Klingström J., Lundkvist A. (2008). Puumala hantavirus excretion kinetics in bank voles (*Myodes glareolus*). Emerg. Inf. Dis..

[33] Schountz T., Shaw T.I., Glenn T.C., Feldmann H., Prescott J. (2013). Expression profiling of lymph node cells from deer mice infected with Andes virus. BMC Immunol..

[34] The Mhc sequencing consortium (1999). Complete sequence and genemap of a human major histocompatibility complex. Nature.

[35] Klein J. (1986). The Natural History of the Major Histocompatibility Complex.

[36] Robinson J., Halliwell J.A., McWilliam H., Lopez R., Parham P., Marsh S.G.E. (2013). The IMGT/HLA Database. Nucl. Acids Res..

[37] Bernatchez L., Landry C. (2003). Mhc studies in nonmodel vertebrates: What have we learned about natural selection in 15 years. J. Evol. Biol..

[38] Mustonen J., Partanen J., Kanerva M., Pietilä K., Vapalahti O., Pasternack A., Vaheri A. (1996). Genetic susceptibility to severe course of nephropathia epidemica caused by Puumala hantavirus. Kidney Int..

[39] Plyusnin A., Hörling J., Kanerva M., Mustonen J., Cheng Y., Partanen J., Vapalahti O., Kukkonen S.K., Niemimaa J., Henttonen H. (1997). Puumala hantavirus genome in patients with nephropathia epidemica: Correlation of PCR positivity with HLA haplotype and link to viral sequences in local rodents. J. Clin. Microbiol..

[40] Mustonen J., Partanen J., Kanerva M., Pietilä K., Vapalahti O., Pasternack A., Vaheri A. (1998). Association of HLA-B27 with benign clinical course of nephropathia epidemica caused by Puumala hantavirus. Scand. J. Immunol..

[41] Korva M., Saksida A., Kunilo S., Jeras B.V., Avsic-Zupanc T. (2011). HLA-associated hemorrhagic fever with renal syndrome disease progression in Slovenian patients. Clin. Vacc. Immunol..

[42] Ma Y., Yuan B., Yi J., Zhuang R., Wang J., Zhang Y., Xu Z., Zhang Y., Liu B., Wei C. (2012). The genetic polymorphisms of HLA are strongly correlated with the disease severity after Hantaan virus infection in the Chinese Han population. Clin. Dev. Immunol..

[43] Koster F., Foucar K., Hjelle B., Scott A., Chong Y.Y., Larson R., McCabe M. (2001). Rapid presumptive diagnosis of hantavirus cardiopulmonary syndrome by peripheral blood smear review. Am. J. Clin. Pathol..

[44] Kilpatrick E.D., Terajima M., Koster F.T., Catalina M.D., Cruz J., Ennis F.A. (2004). Role of specific CD8(+) T cells in the severity of a fulminant zoonotic viral hemorrhagic fever, hantavirus pulmonary syndrome. J. Immunol..

[45] Terajima M., Ennis F.A. (2011). T cells and pathogenesis of hantavirus cardiopulmonary syndrome and hemorrhagic fever with renal syndrome. Viruses.

[46] Manigold T., Mori A., Graumann R., Llop E., Simon V., Ferres M., Valdivieso F., Castillo C., Hjelle B., Vial P. (2010). Highly differentiated, resting Gn-specific memory CD8 + T cells persist years after infection by Andes hantavirus. PLoS Pathog..

[47] Ferrer C.P., Vial C.P.A., Ferres G.M., Godoy M.P., Cuiza V.A., Marco C.C., Castillo H.C., Umana C.M.E., Rothhammer E.F., Llop R.E. (2007). Genetic susceptibility to Andes hantavirus: Association between severity of disease and HLA alleles in Chilean patients. Revist. Chilena Infect..

[48] Wang M.L., Lai J.H., Zhu Y., Zhang H.B., Li C., Wang J.P., Li Y.M., Yang A.G., Jin B.Q. (2009). Genetic susceptibility to haemorrhagic fever with renal syndrome caused by Hantaan virus in Chinese Han population. Int. J. Immunogenet..

[49] Candore G., Cigna D., Gervasi F., Colucci A.T., Modica M.A., Caruso C. (1994). *In vitro* cytokine production by hla-b8,dr3 positive subjects. Autoimmunity.

[50] Rudwaleit M., Siegert S., Yin Z., Eick J., Thiel A., Radbruch A., Sieper J., Braun J. (2001). Low T cell production of TNF-alpha and IFN-gamma in ankylosing spondylitis: Its relation to HLA-B27 and influence of the TNF-308 gene polymorphism. Ann. Rheum. Dis..

[51] Kanerva M., Vaheri A., Mustonen J., Partanen J. (1998). High-producer allele of tumour necrosis factor-alpha is part of the susceptibility MHC haplotype in severe Puumala virus-induced nephropathia epidemica. Scand. J. Inf. Dis..

[52] Temonen M., Mustonen J., Helin H., Pasternack A., Vaheri A., Holthofer H. (1996). Cytokines, adhesion molecules, and cellular infiltration in nephropathia epidemica kidneys: An immunohistochemical study. Clin. Immunol. Immunopath..

[53] Maes P., Clement J., Groeneveld P.H.P., Colson P., Huizinga T.W.J., Van Ranst M. (2006). Tumor necrosis factor-alpha genetic predisposing factors can influence clinical severity in nephropathia epidemica. Viral Immunol..

[54] Maes P., Clement J., Gavrilovskaya I., Van Ranst M. (2004). Hantaviruses: Immunology, treatment, and prevention. Viral Immunol..

[55] Borges A.A., Donadi E.A., Campos G.M., Moreli M.L., de Sousa R.L.M., Saggioro F.P., de Figueiredo G.G., Badra S.J., Deghaide N.H.S., Figueiredo L.T.M. (2010). Association of-308G/A polymorphism in the tumor necrosis factor-alpha gene promoter with susceptibility to development of hantavirus cardiopulmonary syndrome in the Ribeiro Preto region, Brazil. Arch. Virol..

[56] Sane J., Laine O., Mäkelä S., Paakkala A., Jarva H., Mustonen J., Vapalahti O., Meri S., Vaheri A. (2012). Complement activation in Puumala hantavirus infection correlates with disease severity. Ann. Med..

[57] Plyusnina A., Razzauti M., Sironen T., Niemimaa J., Vapalahti O., Vaheri A., Henttonen H., Plyusnin A. (2012). Analysis of complete Puumala virus genome, Finland. Emerg. Inf. Dis..

[58] Mäkelä S., Hurme M., Ala-Houhala I., Mustonen J., Koivisto A.M., Partanen J., Vapalahti O., Vaheri A., Pasternack A. (2001). Polymorphism of the cytokine genes in hospitalized patients with Puumala hantavirus infection. Nephrol. Dial. Transpl..

[59] Liu Z., Gao M., Han Q., Lou S., Fang J. (2009). Platelet glycoprotein iib/iiia (HPA-1 and HPA-3) polymorphisms in patients with hemorrhagic fever with renal syndrome. Hum. Immunol..

[60] Laine O., Joutsi-Korhonen L., Mäkelä S., Mikkelsson J., Pessi T., Tuomisto S., Huhtala H., Libraty D., Vaheri A., Karhunen P. (2012). Polymorphisms of PAI-1 and platelet GP-ia may associate with impairment of renal function and thrombocytopenia in Puumala hantavirus infection. Thromb. Res..

[61] Baigil’dina A.A., Islamgulov D.V. (2012). Genetic determining of the change in VE-cadherin expression and intensified vessel deendothelisation during hemorrhagic fever with renal syndrome. Mol. Genet. Microbiol. Virol..

[62] Mäkelä S., Mustonen J., Ala-Houhala I., Hurme M., Koivisto A.M., Vaheri A., Pasternack A. (2004). Urinary excretion of interleukin-6 correlates with proteinuria in acute Puumala hantavirus-induced nephritis. Am. J. Kidney Dis..

[63] Libraty D.H., Mäkelä S., Vlk J., Hurme M., Vaheri A., Ennis F.A., Mustonen J. (2012). The degree of leukocytosis and urine gata-3 mRNA levels are risk factors for severe acute kidney injury in Puumala virus nephropathia epidemica. PLoS One.

[64] Outinen T.K., Mäkelä S.M., Ala-Houhala I.O., Huhtala H.S.A., Hurme M., Paakkala A.S., Porsti I.H., Syrjänen J.T., Mustonen J.T. (2010). The severity of Puumala hantavirus induced nephropathia epidemica can be better evaluated using plasma interleukin-6 than C-reactive protein determinations. BMC Inf. Dis..

[65] Sadeghi M., Eckerle I., Daniel V., Burkhardt U., Opelz G., Schnitzler P. (2011). Cytokine expression during early and late phase of acute Puumala hantavirus infection. BMC Immunol..

[66] Kyriakidis I., Papa A. (2013). Serum TNF-alpha, STNFR1, IL-6, IL-8 and IL-10 levels in hemorrhagic fever with renal syndrome. Virus Res..

[67] Liu Z., Gao M., Han Q., Fang J., Zhao Q., Zhang N. (2008). Intensity of platelet beta (3) integrin in patients with hemorrhagic fever with renal syndrome and its correlation with disease severity. Virus Immunol..

[68] Ma Y., Liu B., Yuan B., Wang J., Yu H., Zhang Y., Xu Z., Zhang Y., Yi J., Zhang C. (2012). Sustained high level of serum VEGF at convalescent stage contributes to the renal recovery after HTNV infection in patients with hemorrhagic fever with renal syndrome. Clin. Dev. Immunol..

[69] Kotlik P., Deffontaine V., Mascheretti S., Zima J., Michaux J.R., Searle J.B. (2006). A northern glacial refugium for bank voles (*Clethrionomys glareolus*). Proc. Nat. Acad. Sci. USA.

[70] Botten J., Mirowsky K., Kusewitt D., Bharadwaj M., Yee J., Ricci R., Feddersen R.M., Hjelle B. (2000). Experimental infection model for Sin Nombre hantavirus in the deer mouse (*Peromyscus maniculatus*). Proc. Nat. Acad. Sci. USA.

[71] Voutilainen L. (2013). Interactions between Puumala Hantavirus and Its Host, the Bank Vole, in the Boreal Zone. Ph.D. Thesis.

[72] Bernshtein A.D., Apekina N.S., Mikhailova T.V., Myasnikov Y.A., Khlyap L.A., Korotkov Y.S., Gavrilovskaya I.N. (1999). Dynamics of Puumala hantavirus infection in naturally infected bank voles (*Clethrionomys glareolus*). Arch. Virol..

[73] Deter J., Chaval Y., Galan M., Gauffre B., Morand S., Henttonen H., Laakkonen J., Voutilainen L., Charbonnel N., Cosson J.F. (2008). Kinship, dispersal and hantavirus transmission in bank and common voles. Arch. Virol..

[74] Mills J.N., Ksiazek T.G., Ellis B.A., Rollin P.E., Nichol S.T., Yates T.L., Gannon W.L., Levy C.E., Engelthaler D.M., Davis T. (1997). Patterns of association with host and habitat: Antibody reactive with Sin Nombre virus in small mammals in the major biotic communities of the southwestern united states. Am. J. Trop. Med. Hyg..

[75] Klein S.L., Cernetich A., Hilmer S., Hoffman E.P., Scott A.L., Glass G.E. (2004). Differential expression of immunoregulatory genes in male and female norway rats following infection with Seoul virus. J. Med. Virol..

[76] Hannah M.F., Bajic V.B., Klein S.L. (2008). Sex differences in the recognition of and innate antiviral responses to seoul virus in norway rats. Brain Behav. Immun..

[77] Botten J., Mirowsky K., Kusewitt D., Ye C.Y., Gottlieb K., Prescott J., Hjelle B. (2003). Persistent sin nombre virus infection in the deer mouse (*Peromyscus maniculatus*) model: Sites of replication and strand-specific expression. J. Virol..

[78] Schountz T., Prescott J., Cogswell A.C., Oko L., Mirowsky-Garcia K., Galvez A.P., Hjelle B. (2007). Regulatory T cell-like responses in deer mice persistently infected with Sin Nombre virus. Proc. Nat. Acad. Sci. USA.

[79] Easterbrook J.D., Zink M.C., Klein S.L. (2007). Regulatory T cells enhance persistence of the zoonotic pathogen Seoul virus in its reservoir host. Proc. Nat. Acad. Sci. USA.

[80] Easterbrook J.D., Klein S.L. (2008). Seoul virus enhances regulatory and reduces proinflammatory responses in male norway rats. J. Med. Virol..

[81] Schonrich G., Rang A., Lutteke N., Raftery M.J., Charbonnel N., Ulrich R.G. (2008). Hantavirus-induced immunity in rodent reservoirs and humans. Immunol. Rev..

[82] Klingström J., Heyman P., Escutenaire S., Sjölander K.B., De Jaegere F., Henttonen H., Lundkvist A. (2002). Rodent host specificity of European hantaviruses: Evidence of Puumala virus interspecific spillover. J. Med. Virol..

[83] Gavrilovskaya I.N., Brown E.J., Ginsberg M.H., Mackow E.R. (1999). Cellular entry of hantaviruses which cause hemorrhagic fever with renal syndrome is mediated by beta3 integrins. J. Virol..

[84] Gavrilovskaya I.N., Peresleni T., Geimonen E., Mackow E.R. (2002). Pathogenic hantaviruses selectively inhibit beta3 integrin directed endothelial cell migration. Arch. Virol. S.

[85] Gavrilovskaya I.N., Shepley M., Shaw R., Ginsberg M.H., Mackow E.R. (1998). Beta3 integrins mediate the cellular entry of hantaviruses that cause respiratory failure. Proc. Nat. Acad. Sci. USA.

[86] Raymond T., Gorbunova E., Gavrilovskaya I.N., Mackow E.R. (2005). Pathogenic hantaviruses bind plexin-semaphorin-integrin domains present at the apex of inactive, bent alphavbeta3 integrin conformers. Proc. Nat. Acad. Sci. USA.

[87] Mou D.L., Wang Y.P., Huang C.X., Li G.Y., Pan L., Yang W.S., Bai X.F. (2006). Cellular entry of Hantaan virus A9 strain: Specific interactions with beta 3 integrins and a novel 70 kda protein. Biochem. Bioph. Res. Co..

[88] Matthys V.S., Gorbunova E.E., Gavrilovskaya I.N., Mackow E.R. (2010). Andes virus recognition of human and syrian hamster beta (3) integrins is determined by an l33p substitution in the PSI domain. J. Virol..

[89] Krautkramer E., Zeier M. (2008). Hantavirus causing hemorrhagic fever with renal syndrome enters from the apical surface and requires decay-accelerating factor (DAF/CD55). J. Virol..

[90] Choi Y., Kwon Y.-C., Kim S.-I., Park J.-M., Lee K.-H., Ahn B.-Y. (2008). A hantavirus causing hemorrhagic fever with renal syndrome requires gc1qr/p32 for efficient cell binding and infection. Virology.

[91] Bagamian K.H., Towner J.S., Kuenzi A.J., Douglass R.J., Rollin P.E., Waller L.A., Mills J.N. (2012). Transmission ecology of Sin Nombre hantavirus in naturally infected North American deer mouse populations in outdoor enclosures. PLoS One.

[92] Lyubsky S., Gavrilovskaya I., Luft B., Mackow E. (1996). Histopathology of *Peromyscus leucopus* naturally infected with pathogenic NY-1 hantaviruses: pathologic markers of HPS viral infection in mice. Lab. Invest..

[93] Netski D., Thran B.H., St Jeor S.C. (1999). Sin Nombre virus pathogenesis in *Peromyscus maniculatus*. J. Virol..

[94] Guivier E., Galan M., Male P.J.G., Kallio E.R., Voutilainen L., Henttonen H., Olsson G.E., Lundkvist A., Tersago K., Augot D. (2010). Associations between MHC genes and Puumala virus infection in *Myodes glareolus* are detected in wild populations, but not from experimental infection data. J. Gen. Virol..

[95] Spurgin L.G., Richardson D.S. (2010). How pathogens drive genetic diversity: Mhc, mechanisms and misunderstandings. Proc. Roy. Soc. Lond. B.

[96] Male P.-J.G., Martin J.-F., Galan M., Deffontaine V., Bryja J., Cosson J.-F., Michaux J., Charbonnel N. (2012). Discongruence of MHC and cytochrome b phylogeographical patterns in *Myodes glareolus* (rodentia: Cricetidae). Biol. J. Linn. Soc..

[97] Rohfritsch A., Guivier E., Galan M., Chaval Y., Charbonnel N. (2013). Apport de l’immunogénétique à la compréhension des interactions entre le campagnol roussâtre *Myodes glareolus* et l’hantavirus Puumala. Bulletin de l'académie vétérinaire de France.

[98] Dupanloup I., Schneider S., Excoffier L. (2002). A simulated annealing approach to define the genetic structure of populations. Mol. Ecol..

[99] Guivier E. (2010). Variabilité de la résistance/tolérance des campagnols roussatres à l’hantavirus Puumala et conséquences épidémiologiques. Ph.D. Thesis.

[100] Deffontaine V., Libois R., Kotlik P., Sommer R., Nieberding C., Paradis E., Searle J.B., Michaux J.R. (2005). Beyond the mediterranean peninsulas: Evidence of central european glacial refugia for a temperate forest mammal species, the bank vole (*Clethrionomys glareolus*). Mol. Ecol..

[101] Nemirov K., Leirs H., Lundkvist A., Olsson G.E. (2010). Puumala hantavirus and *Myodes glareolus* in northern Europe: No evidence of co-divergence between genetic lineages of virus and host. J. Gen. Virol..

[102] Deter J., Chaval Y., Galan M., Henttonen H., Laakkonen J., Voutilainen L., Ribas Salvador A., Bryja J., Morand S., Cosson J.F. (2008). Association between the DQA MHC class II gene and Puumala virus infection in the specific reservoir *Myodes glareolus*, the bank vole. Inf. Genet. Evol..

[103] Ribas Salvador A., Guivier E., Xuereb A., Chaval Y., Cadet P., Poulle M.L., Sironen T., Voutilainen L., Henttonen H., Cosson J.F. (2011). Concomitant influence of helminth infection and landscape on the distribution of Puumala hantavirus in its reservoir, *Myodes glareolus*. BMC Microbiol..

[104] Guivier E., Galan M., Ribas Salvador A., Xuéreb A., Chaval Y., Olsson G., Essbauer S., Henttonen H., Voutilainen L., Cosson J.F. (2010). *Tnf-α* expression and promoter sequences reflect the balance of tolerance/resistance to Puumala virus infection in European bank vole populations. Inf. Genet. Evol..

[105] Haldane J.B.S. (1956). The estimation and significance of the logarithm of a ratio of frequencies. Ann. Hum. Genet..

[106] Råberg L., Graham A.L., Read A.F. (2009). Decomposing health: Tolerance and resistance to parasites in animals. Philos. Trans. Roy. Soc. Lond. B.

[107] Jin H.K., Yoshimatsu K., Takada A., Ogino M., Asano A., Arikawa J., Watanabe T. (2001). Mouse Mx2 protein inhibits hantavirus but not influenza virus replication. Arch. Virol..

[108] Li Y., Youssoufian H. (1997). Mxa overexpression reveals a common genetic link among four fanconi anemia complementation groups. J. Clin. Investig..

[109] Porter B.F., Ambrus A., Storts R.W. (2006). Immunohistochemical evaluation of Mx protein expression in canine encephalitides. Vet. Pathol..

[110] Guivier E., Galan M., Chaval Y., Xuereb A., Ribas Salvador A., Poulle M.L., Charbonnel N., Cosson J.F. (2011). Landscape genetics highlights the role of bank vole metapopulation dynamics in the epidemiology of Puumala hantavirus. Mol. Ecol..

[111] The International HapMap Consortium (2010). Integrating common and rare genetic variation in diverse human populations. Nature..

[112] The 1000 Genomes Project Consortium (2012). An integrated map of genetic variation from 1092 human genomes. Nature..

[113] Barreiro L.B., Patin E., Neyrolles O. (2005). The heritage of pathogen pressures and ancient demography in the human innate-immunity CD209/CD209l region. Am. J. Hum. Genet..

[114] Hedrick P.W. (2012). Resistance to malaria in humans: The impact of strong, recent selection. Malaria J..

[115] Schountz T., Acuna-Retamar M., Feinstein S., Prescott J., Torres-Perez F., Podell B., Peters S., Ye C., Black W.C., Hjelle B. (2012). Kinetics of immune responses in deer mice experimentally infected with Sin Nombre virus. J. Virol..

[116] Schneider D.S., Ayres J.S. (2008). Two ways to survive infection: What resistance and tolerance can teach us about treating infectious diseases. Nat. Rev. Immunol..

[117] Lloyd-Smith J.O., Schreiber S.J., Kopp P.E., Getz W.M. (2005). Superspreading and the effect of individual variation on disease emergence. Nature.

[118] Stein R.A. (2011). Super-spreaders in infectious diseases. Int. J. Inf. Dis..

[119] Klempa B., Witkowski P.T., Popugaeva E., Auste B., Koivogui L., Fichet-Calvet E., Strecker T., ter Meulen J., Krueger D.H. (2012). Sangassou virus, the first hantavirus isolate from Africa, displays genetic and functional properties distinct from those of other murinae-associated hantaviruse. J. Virol..

[120] Milazzo M.L., Eyzaguirre E.J., Molina C.P., Fulhorst C.F. (2002). Maporal viral infection in the syrian golden hamster: A model of hantavirus pulmonary syndrome. J. Infect. Dis..

[121] Safronetz D., Ebihara H., Feldmann H., Hooper J.W. (2012). The syrian hamster model of hantavirus pulmonary syndrome. Antivir. Res..

[122] Klempa B., Avsic-Zupanc T., Clement J., Dzagurova T.K., Henttonen H., Heyman P., Jakab F., Kruger D.H., Maes P., Papa A. (2013). Complex evolution and epidemiology of Dobrava-Belgrade hantavirus: Definition of genotypes and their characteristics. Arch. Virol..

[123] Mackow E.R., Gavrilovskaya I.N. (2001). Cellular receptors and hantavirus pathogenesis. Hantaviruses.

[124] Schultze D., Lundkvist Å., Blauenstein U., Heyman P. (2002). Tula virus infection associated with fever and exanthema after a wild rodent bite. Eur. J. Clin. Microbiol. Infect. Dis..

[125] Klempa B., Koivogui L., Sylla O., Koulemou K., Auste B., Ulrich K., Kruger D.H., Meulen J. (2010). Serological evidence of human hantavirus infections in Guinea, West Africa. J. Inf. Dis..

[126] Zelená H., Mrázek J., Kuhn T. (2013). Tula hantavirus infection in immunocompromised host, Czech Republic. Emerg. Infect. Dis..

